# Towards Characterizing and Developing Formation and Migration Cues in Seafloor Sand Waves on Topology, Morphology, Evolution from High-Resolution Mapping via Side-Scan Sonar in Autonomous Underwater Vehicles

**DOI:** 10.3390/s21093283

**Published:** 2021-05-10

**Authors:** Rui Nian, Lina Zang, Xue Geng, Fei Yu, Shidong Ren, Bo He, Xishuang Li

**Affiliations:** 1 School of Information Science and Engineering, Ocean University of China, Qingdao 266000, China; zanglina0103@163.com (L.Z.); gengxue06@163.com (X.G.); yf0327sky@163.com (F.Y.); rendong1996@gmail.com (S.R.); bhe@ouc.edu.cn (B.H.); 2 Key Laboratory of Marine Geology and Metallogeny, Ministry of Nature Resources of People’s Republic of China, Qingdao 266061, China; lxs@fio.org.cn

**Keywords:** side scan sonar, seafloor sand waves, echo intensity, sand waves migration, geometrical morphology topology of sand waves, zero crossing rate

## Abstract

Sand waves constitute ubiquitous geomorphology distribution in the ocean. In this paper, we quantitatively investigate the sand wave variation of topology, morphology, and evolution from the high-resolution mapping of a side scan sonar (SSS) in an Autonomous Underwater Vehicle (AUV), in favor of online sequential Extreme Learning Machine (OS-ELM). We utilize echo intensity directly derived from SSS to help accelerate detection and localization, denote a collection of Gaussian-type morphological templates, with one integrated matching criterion for similarity assessment, discuss the envelope demodulation, zero-crossing rate (ZCR), cross-correlation statistically, and estimate the specific morphological parameters. It is demonstrated that the sand wave detection rate could reach up to 95.61% averagely, comparable to deep learning such as MobileNet, but at a much higher speed, with the average test time of 0.0018 s, which is particularly superior for sand waves at smaller scales. The calculation of morphological parameters primarily infer a wave length range and composition ratio in all types of sand waves, implying the possible dominant direction of hydrodynamics. The proposed scheme permits to delicately and adaptively explore the submarine geomorphology of sand waves with online computation strategies and symmetrically integrate evidence of its spatio-temporal responses during formation and migration.

## 1. Introduction

The submarine sand wave, one type of ubiquitous geomorphology distributed widely in the ocean, is highly correlative with multivariate marine environmental factors in their formation and migration process [1,2,3,4]. It is of great research value to understand sand wave dynamics in both marine science and engineering, such as the construction assessment of submarine pipelines and oil platforms [5,6]. To quantitatively identify sand wave variation in its topology, morphology, and evolution over time could symmetrically integrate evidence of its spatio-temporal responses to manifold intrinsic and external indices and help provide opportunities to explore the underlying patterns and mechanisms of their formation and migration.

Generally, submarine sand waves exhibit at a variety of scales, from small-scale megaripples to large-scale sand banks [1,7], which is actually a reflection of the variation under the influence of hydrodynamic forces [8], closely relevant to the coupling impact factors, such as the tidal characteristics, sediment grain sizes, the related residual currents, and so forth [9,10,11,12]. For decades, researchers have been working on topics with regard to the formation, detection, migration, simulation for submarine sand waves [2,5,13,14,15,16,17,18]. The existing techniques could be roughly divided into the numerical methods [5,13], mathematical modeling [19,20], field survey [21,22], remote sensing [18,23], and so forth. The classical numerical approaches could be performed by simple formulas and convenient calculation [13]. However, such empirical calculation tends to be composed of multiple parameters that must be considered, which would be sometimes very difficult to derive, and thereby possibly restrict the accuracy and applicable ranges; hence, often a rough estimation is used. The mathematical modeling could explain the generation and predict the migration of sand waves in an efficient and economical way [2,15], but no models could be universally applied. Remote sensing provides one highly efficient and dynamically observable means to conduct large-scale regional surveys of sand waves [18], but might lack showing morphological details in sand waves at small scales. The field surveys mainly employ the acoustic equipment, such as the multibeam bathymetry system, side scan sonar, sub-bottom profiler, and acoustic Doppler current profiler (ADCP), and so on to conduct sand wave surveys on site, which enables more specific investigation in the target underwater region at a higher accuracy [5,14,22]. However, usually this comes with a high cost as well as restrictions of availability by the marine environment conditions and submarine topography.

Recently, there has been increasing attention paid to the deployment of Autonomous Underwater Vehicles (AUVs) and Remote Operated Vehicles (ROV) in the field survey of submarine sand waves [24,25], e.g., Bluefin [26], Hugin [27], Remus [28], Iver2 [29], JAMSTEC [30], and so forth, which could greatly break through the restrictions of the traditional field survey driven by research vessels on a planned voyage, with more flexibility and adaptivity in applications. Underwater vehicles could be equipped with a series of sensors to complete a variety of sand wave inspection tasks and proceed at a lower speed and at a shallower depth towards the seafloor, allowing the morphological imagery at a higher resolution. AUVs are capable of carrying out a lawnmower pattern, i.e., a set of overlapping strips, which permits submarine geomorphology delicately across a certain range of underwater region rather than only one long narrow swath.

Among those categories of acoustic sensors, the side scan sonar (SSS) is one of the most widely mounted sensors on AUVs for sand wave inspections, due to its portability and imaging capacity. When the AUV proceeds along its track, the SSS emits acoustic waves to construct seafloor mapping for sand waves with the varied echo intensities. The direct use of SSS imaging could basically bear a fairly high coverage rate of seafloor mapping with time. However, acoustic echoes from SSS could be very easily interfered by various kinds of environmental noise, reverberation noise, instrument noise during their propagating process in the ocean, there will be a lot of high-frequency noises included in the waveform of sand waves, which makes it quite challenging to distinguish functional signals. Synthesizing consecutive uniform pings of echo intensities and converting these into SSS imaging might initially bring possible distortion, stretches, and compression to some extent, especially when the attitude of AUVs (roll, pitch, yaw) changes over time. The configuration of relatively poor real-time computational abilities in most AUVs may restrict online detection for sand waves, so in most cases, it still remains difficult to identify sand waves and explore the morphological and topological parameters on site from SSS imaging collected by AUVs simultaneously with high accuracy and efficiency. Hence, here we make an attempt to utilize echo intensities directly derived from SSS to help AUVs accelerate the online detection and localization process for sand waves.

So far, machine learning has been regarded as one of the most powerful models in exploring seafloor morphological and topological properties that could discriminate and approximate nonlinear function without prior assumptions. All kinds of advanced algorithms, particularly the deep learning framework, have been developed and led to progress in the understanding of SSS imaging [31,32,33]. However, the use of such deep learning algorithms could raise higher requirement to the super-computing capacities, such as high-performance GPUs, and might suffer from insufficient learning, especially when new observations arrive progressively [34,35,36]. Recently, the extreme learning machine (ELM) has attracted more and more attention by providing comparable performances with simplified architecture at a much faster speed [37,38]. The online sequential extreme learning machine (OS-ELM) inherits the merits of ELM and bears an online learning ability when observations come in succession [39], which is more suitable for few shot learning in sand wave detection and overcomes the incomplete or unstructured data acquisition by AUVs [40,41].

Meanwhile, several previous studies [42,43,44,45] have proved that the sand wave attributes reflected from the acoustic cross-section in SSS imaging could be utilized as the morphological and topological indicators of sand wave dynamics during the evolution process of the seabed load sediment transport under hydrodynamic conditions. The asymmetric shape and orientation of sand waves would reveal the ebb flow dominance in the navigation channel, and sand wave size and spacing have been analyzed, both in natural environments [46], in laboratories [47], and through morphological modeling [19]. The hydro-sedimentological conditions could be also inferred with the help of morphometric relationships between sand waves. Wynn et al. [48] used a combination of high-resolution SSS and seafloor photography to image barchan dunes and sand ripples in the Faroe-Shetland Channel and have shown that high-resolution imaging of seafloor bedforms could provide some indication of the bottom-current orientation and flow velocity, and the bottom currents are modified by dune morphology. Dijk et al. [49] determined the morphology and dynamics of sand waves from time records of SSS and multibeam imagery and found that the coastal and offshore compound sand waves were nonrelic forms that actively develop and migrate. Passchier et al. [50] investigated the influences of seasonal storm events and fair-weather periods on the stability of large-scale compound sand wave forms by SSS off the Dutch coast, and they found that multiple seasonal storm events of low intensity did not have a measurable effect on sand wave morphology or position. Crawford et al. [51] extracted seabed sand wave orientation, wavelength, and defect density parameters by a novel image processing technique based on fingerprint analysis from SSS.

In this paper, starting from the inspiration to quantitatively investigate the geomorphology in sand wave profiles along a AUV’s track, we directly extract the echo intensity of each ping from SSS imaging through time varying gain (TVG) correction, speed correction, blind zone removal, and ensemble empirical mode decomposition (EEMD) denoising to compensate the frequent motion variation in an AUV as well as the noise interference, energy attenuation, and multi-path effects over time. Both ELM learning and some classical approaches have been jointly employed and aim to potentially provide a mutually reinforcing and complementary, related solution. We establish OS-ELM architecture and feed into the echo intensity sub-sequences as inputs, respectively, from the historical survey with manually labeled annotation, as well as the online observation that newly arrives at any time, in the context of few shot learning, to accelerate online detection and localization for sand waves. Model selection is involved by assessing performances across the configuration of hyper parameters, and the developed scheme will be comprehensively evaluated by the detection accuracy, False Negative Rate (FNR), False Positive Rate (FPR), and F-Score. We propose to utilize a collection of Gaussian-type morphological templates and construct one combination of matching criterion for similarity assessment in terms of cross-correlation and RMSE through the Taylor formula. We repeatedly deployed our AUV system around Jiaozhou Bay, Qingdao, China in 2019 and 2021, within the duration of more than one year, equipped with SSS. The basic parameters describing the morphological characteristics of sand waves will be elaborately discussed and finely grained in the study region, with the help of the statistics in the envelope demodulation, zero-crossing rate (ZCR) spectrum, and the cross-correlation coefficient; and the specific morphological parameters, including wave length, wave height, asymmetric index, will be further estimated, so as to explore the topology, morphology, and evolution in sand waves at a higher resolution with online computation strategies and provide insights into the spatial-temporal evidence in the formation and migration process.

The remainder of the paper is organized as follows: Section 2, Section 3 and Section 4 respectively describe the basic principle of the side scan sonar, the basics in ELM learning, and template matching. Section 5 introduces the sand wave online detection with OS-ELM and their morphological, geometrical, and topological characterization by echo intensity. Section 6 shows the simulation experiment and result analysis. Section 7 discusses the envelope demodulation, zero-crossing rate spectrum, cross-correlation coefficients, and the specific morphological parameters including wave length, wave height, and asymmetric index, by means of echo intensity in the SSS profile.

## 2. Side Scan Sonar Principle

**Figure 1:**
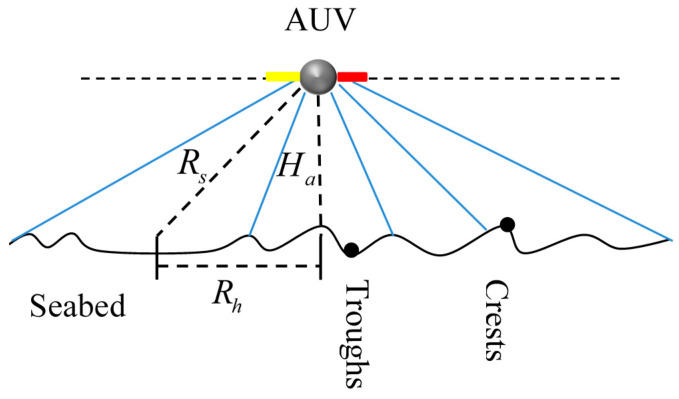
SSS characterization in sand wave inspections.

The side scan sonar (SSS) is the one of the most basic acoustic sensors employed by AUVs during marine investigation tasks. When our AUV sails forward, the SSS transmits and receives echoes at certain time intervals, then displays as the echo intensity, and then composes the acoustic imaging for the seafloor sand waves. The transducers emit an acoustic pulse with directivity. When it reaches the seabed, the reflected waves or the backscattered waves return back along the original route, and the near echo first reach the transducers. In general, the hard, rough, and protuberant seabed could have strong echoes, while the soft, flat, and concave seabed will correspond to weak echoes. The concave sand waves at the bottom obscured by the crests could receive no echoes, yielding the shadow zone. Furthermore, the echo amplitude increases at the protuberant crest. Therefore, the echo amplitude reflects the ups and downs on seafloor sand waves, as is shown in Figure 1.

The spatial relationship between the operating range and AUV height is,

(1) $$R_{h}=\sqrt{R_{s}{}^{2}-H_{a}{}^{2}}$$

where $R_{h}$ is the operating range per side, and $R_{s}$ is the slant range. $H_{a}$ is the AUV height above seabed.

## 3. Basic ELM

So far, ELM learning has been developed to work at a much faster learning speed with a higher generalization performance in the pattern recognition. For the given N learning samples ${\left\{x_{i},y_{i}\right\}}_{i=1}^{N}$, where $x_{i}={\left[x_{i1},x_{i2},\ldots ,x_{in}\right]}^{T}$ and $y_{i}={\left[y_{i1},y_{i2},\ldots ,y_{im}\right]}^{T}$, the standard model of the ELM learning can be written as the following matrix format:

(2) $$\begin{array}{l} H\beta =Y\\ H(x)=[h_{1}h_{2}\cdots h_{L}]\\ =\left[\begin{array}{l} h_{1}(x_{1})\cdots h_{L}(x_{1})\\ \;\;\;\vdots \;\;\;\;h_{i}(x_{j})\;\;\;\;\vdots \\ h_{1}(x_{N})\cdots h_{L}(x_{N}) \end{array}\right]\\ ={\left[\begin{array}{l} \varphi (w_{1}\times x_{1}+b_{1})\cdots \varphi (w_{L}\times x_{1}+b_{L})\\ \;\;\;\;\;\;\vdots \;\;\;\;\;\;\varphi (w_{i}\times x_{j}+b_{i})\;\;\;\;\;\;\;\vdots \\ \varphi (w_{1}\times x_{N}+b_{1})\cdots \varphi (w_{L}\times x_{N}+b_{L}) \end{array}\right]}_{N\times L}\\ \beta ={\left[\beta_{1},\beta_{2},\ldots ,\beta_{L}\right]}_{m\times L}^{T},Y={\left[y_{1},y_{2},\ldots ,y_{N}\right]}_{m\times N}^{T} \end{array}$$

where $w_{i}={\left[w_{i1},w_{i2},\ldots ,w_{ir}\right]}^{T},i=1,2,\ldots L$ is the weight vector connecting the ith hidden neuron and the input neurons, $\beta_{i}={\left[\beta_{i1},\beta_{i2},\ldots ,\beta_{im}\right]}_{}^{T}$ is the weight vector connecting the ith hidden neuron and the output neurons, $w_{i}\times x_{j}$ denotes the inner product of $w_{i}\;\mathrm{and}\;x_{j},j=1,2,\ldots N$, and there are L hidden neurons with the activation function φ(x) All kinds of activation functions can be chosen here, such as the Sigmoid function, the hard-limit function, the Gaussian function, and the multiquadric function.

If the activation function φ(x), w and b are all set, the only learning parameter will be β. Different from the traditional learning algorithm, ELM tends to achieve the least training error and the least norm of output weight together. According to Bartlett׳s theory [52], when the feedforward neural networks obtain smaller training errors, the norms of weights are smaller, and the generalization performance of the networks is better, $\beta =\arg\min(||H\beta -Y|{|}^{2},||\beta ||)$. In order to solve the formation, both the standard optimization method and the minimal norm least square method need to be adopted. The original implementation of ELM will then be $\beta =H^{+}Y$, where $H^{+}$ denotes the Moore–Penrose generalized inverse of matrix H [37]. The orthogonal projection method can be used here when $H^{T}H$ is nonsingular and $H^{+}={\left(H^{T}H\right)}^{-1}H^{T}$, or when $HH^{T}$ is nonsingular and $H^{+}=H^{T}{\left(H^{T}H\right)}^{-1}$. In addition, the resulting solution tends to be more stable with a better generalization performance by adding a positive value to the diagonal of $HH^{T}$ or $H^{T}H$ [53].

## 4. Template Matching

The template matching process involves shifting the template over the search area and computing the similarity between the template and the window in the search area over which the template lies. These two steps are described in detail below: (1) Identify the features underlying the search area and build the parameterized template waveform. (2) Match the search area with the parameterized template waveform by setting up an optimization matching criterion. The major similarity measures are the Root Mean Squared Error (RMSE) and the cross-correlation coefficient.

Let $X={\left[x_{1},x_{2},x_{m}\cdots x_{M}\right]}_{1\times M}$ be the search area, and $T={\left[t_{1},t_{2},t_{m},\cdots t_{M}\right]}_{1\times M}$ be the template, and the similarity measurement is by RMSE J(X,T) to screen out the best matches.

(3) $$J(X,T_{})=\sqrt{\sum_{m=1}^{M}{\left(x_{m}^{}-t_{m}\right)}^{2}/M}$$

Cross-correlation coefficient r(X,T) is taken to measure the overall similarity between waveforms and template

(4) $$r(X,T)=Cov(X,T)/\sqrt{D(X)\cdot D(T)}\;\begin{array}{ll} C\mathrm{ov}(X,T) & =E[(X-E\left(X\right))(T-E\left(T\right))]\\ & =E\left[XT\right]-E\left[X\right]E\left[T\right] \end{array}$$

where Cov(X,T) is the covariance of the search area X and template T, D(X) and D(T) stand for the variance of search area X and template T respectively, E[⋅] represents the mathematical expectation.

## 5. Sand Wave Detection and Morphological, Geometrical, Topological Characterization with Echo Intensity

**Figure 2:**
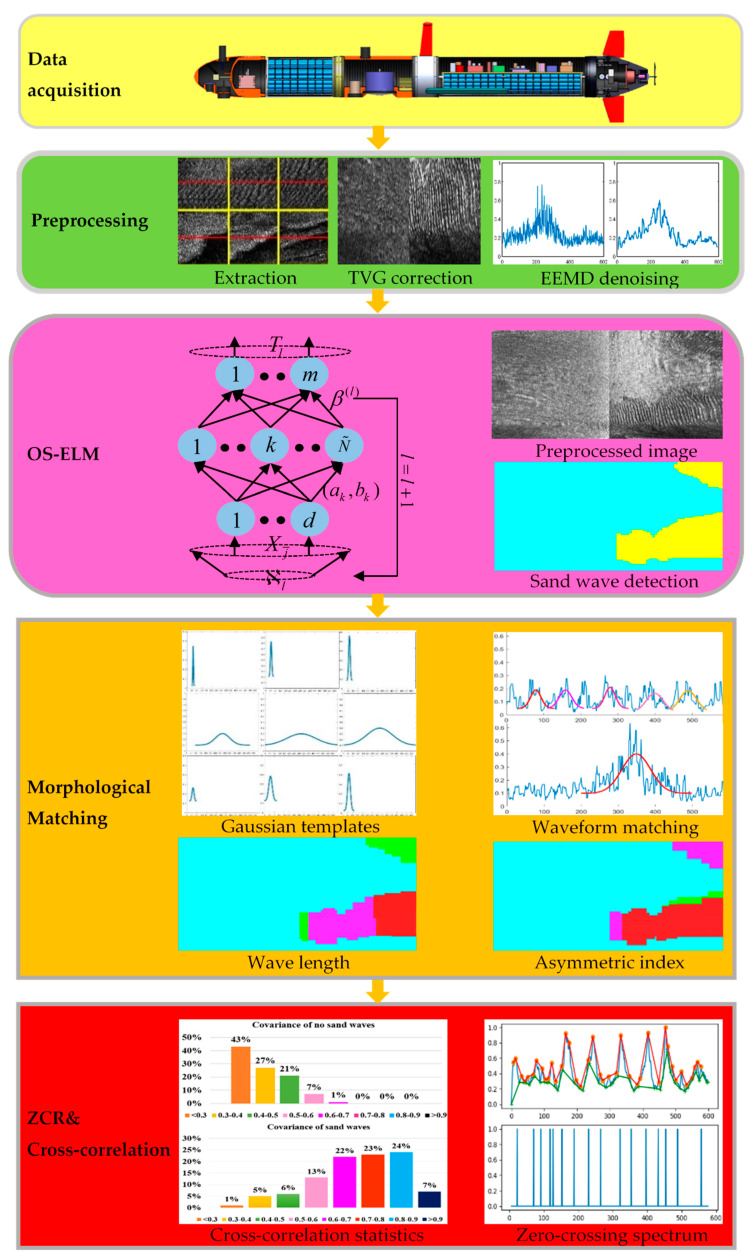
The flow chart of the proposed scheme.

We try to develop the sand wave online detection scheme from the echo intensity in high-resolution SSS mapping via OS-ELM and quantitatively assess the morphological, topological, and geometrical characterization. A brief flow chart of our proposed strategy is shown in Figure 2, which is made up of several correlative steps, including SSS imaging acquisition and preprocessing, sand wave online detection, morphological, geometrical, topological characterization, and the analysis of morphological parameters. Firstly, time varying gain (TVG), speed correction, and ensemble empirical mode decomposition (EEMD) denoising help to compensate the frequent motion in an AUV. We then explore online detection and localization for submarine sand waves through echo intensity, with the help of the OS-ELM learning framework. Furthermore, we denote a collection of Gaussian-type morphological templates, with one integrated matching criterion for similarity assessment. Finally, we statistically discuss the envelope demodulation, zero-crossing rate (ZCR) spectrum, and cross-correlation coefficient and estimate the specific morphological parameters, including wave length, wave height, and asymmetric index.

### 5.1. Echo Waveform Preprocessing

#### 5.1.1. Echo Waveform Extraction

In practice, there are actually quite a few challenges in sand wave inspections via SSS for AUVs. Besides the common problem such as noise interfere and high computational demand, sand waves with a smaller wave length, especially megaripples, tend to be very difficult to distinguish. In addition to SSS imaging, the echo intensity directly reflects the nature of the seafloor with the corresponding geoacoustical properties, and the received backscattered signals are functions of both the geometry of the rough interface in sand waves and the material properties of the ocean bottom, including the density and sound speed ratios as well as the attenuation that is due to the dissipation of acoustic energy through the interface. If we start from the analysis of the sand wave profile instead of only the entire SSS imaging, it would be more beneficial to identify the ridge lines and valley lines for sand waves, infer the direction of sand wave movement, and calculate the sand wave migrate rate, with the advantages of low calculation cost and good real-time performance. Additionally, some smaller sand waves, such as megaripples, might be more easily finely detected after each ping in much detail. We hereby try to explore an alternative scheme for sand wave detection, by utilizing the side scan lines of echo intensity instead of only the mosaicked SSS imaging to accelerate the detection and localization of sand waves.

#### 5.1.2. Time Varying Gain Correction

Acoustic waves experience spreading and absorption losses when traveling through sea water. The further the submarine sand waves are from the SSS transducer, the weaker the echoes we would receive. The horizontal distortion of echo intensity often appears, characterized with higher values in the center region and are smaller than the expected value in the edges. The relationship between echo attenuation and propagation distance can be constructed by propagation loss and reverberation level. One kind of time varying gain (TVG) correction is hereby taken to compensate acoustic attenuation over the distance for echo intensity in each ping as follows.

(5) $$x^{i}{}_{v_{TVG}}=\sqrt{TVG(v)/TVG_{\max}}\times x^{i}{}_{v}$$

where $X_{}^{i}=[x_{}^{i}{}_{1},x_{}^{i}{}_{2},x_{}^{i}{}_{v},\cdots x_{}^{i}{}_{V}]$ is the ith ping of the echo intensity curve, consisting of multiple echo intensity values, v∈[1,w] is defined as the vth position of the echo intensity value along one ping, with w the total width of the echo intensity ping, $x^{i}{}_{v}$ is the vth echo intensity value in one ping, $x^{i}{}_{v_{TVG}}$ is the echo intensity value after TVG correction, and the function TVG(v) [54,55] is denoted as,

(6) $$TVG(v)=A\times \log(C_{v})+B\times (C_{v})\;C_{v}=R_{s}\times |w/2-v|/\left(w/2\right)$$

where the correction coefficient A and the absorption coefficient B are related to the beam shape as well as the emission frequency of acoustic waves, and so forth, which is adjustable according to the scenarios to be applied, $C_{v}$ is the actual seabed projection distance between the current AUV waypoint and the vth echo intensity location, $R_{s}$ is the slant length of the sonar, and w is the width of echo intensity.

#### 5.1.3. Speed Correction

The consecutive pings of echo intensity constitute SSS imaging, which are initially mapped one by one, as if the distance between adjoining pings were uniform. Due to the pose changes, or when the AUV does not keep to a constant speed and route, AUVs possibly experience longitudinal motion instability accompanied by severely fluctuating heave and pitch. The pings sometimes are not evenly and regularly spaced, leading to significant geometrical distortions in the along-track directions. If the AUV slows down, the sand waves in smaller size might look enwidened and enlarged. On the other hand, when speeding up the AUV, it might shrink or even conceal sand waves. Furthermore, if the AUV moves at too high speeds, it can leave unscanned regions in the seafloor that are reflected by echo intensity black gaps. The speed correction is hereby taken to align to the true proportion of sand wave features and fill in the gaps by interpolation. Let $∆h_{i}$ be the voyage distance of each ping data in the side scan sonar image, which could be denoted as [54]:

(7) $$∆h_{i}=\frac{w({\bar{v}}_{i}+{\bar{v}}_{i+1})∆t_{i}}{4R_{s}}=\frac{({\bar{v}}_{i}+{\bar{v}}_{i+1})∆t_{i}}{2}\times \frac{w}{2R_{s}}$$

where $({\bar{v}}_{i}+{\bar{v}}_{i+1})∆t_{i}/2$ refers to the actual distance of AUV in the along-track directions, and $w/2R_{s}$ represents the mapping coefficient between the width of the echo intensity and the actual across-track distance in the seabed, $∆t_{i}$ is the time difference between the ith ping and the (i+1)th ping that the SSS receives echoes, ${\bar{v}}_{i}$ is the instantaneous sailing speed of AUV in the ith ping, ${\bar{v}}_{i+1}$ is the instantaneous speed of AUV in the (i+1)th ping, $R_{s}$ is the slant length of the sonar, w is the width of echo intensity in the ith ping.

#### 5.1.4. EEMD Denoising

Due to the existence of the environmental noise, reverberation noise, and instrument noise in the echo intensity of SSS imaging, recovering the signal with proper filtering is always essential. Frequently, classical filtering methods, such as the Wiener filtering, Gaussian filtering, and mean filtering, have been widely used, but these are still challenging when encountering sharp signals and impulses of short duration for the nonlinear and often nonstationary type.

We therefore employ the Ensemble Empirical Mode Decomposition (EEMD) [56,57] as a self-adaptive filtering for echo intensity denoising, which could adaptively decompose the nonlinear and nonstationary waveform into the sum of components, the Intrinsic Mode Functions (IMFs) and one residual component, and then distinguish and remove IMF with noise as its main component, eliminating the mode mixing problem by adding finite white noise to the investigated signal. The brief process of EEMD denoising [58,59] is expressed as follows:

(1) Add a white noise with the given amplitude to the original signal in echo intensity;
(2) Perform EMD [60] to the signal in echo intensity with the added white noise to obtain P Intrinsic Mode Function (IMF) components and one residual component, whereas the definition and acquisition process of IMF are relegated to Appendix A;
(3) Repeat with the given number of trials. In every trial, the number of IMF P, is a constant. All the trials could be written as,
  (8) $$X_{}^{i}+n_{s}=\sum_{p=1}^{P}IMF_{sp}+r_{s}$$
  where $X_{}^{i}$ is the ith original signal in echo intensity; $n_{s}$is the added white noise of the sth trial; $IMF_{sp}$ is the pth IMF component of the sth trial; $r_{s}$ is the residual component of the sth trial;
(4) Calculate the ensemble mean of all trials and the final decomposition result of the original signal $X_{}^{i}$ in echo intensity by EEMD could be expressed as,
  (9) $$\begin{array}{c} IMF_{p}=\sum_{s=1}^{S}IMF_{sp}/S\\ r(t)=\sum_{s=1}^{S}r_{s}/S\\ X_{}^{i}=\sum_{p=1}^{P}IMF_{p}+r \end{array}$$
  where S is the number of trials, and r is the residual component of EEMD;
(5) To eliminate the noise and maintain the integrity of useful information, the first $p_{1}$ IMFs are selected to be processed with the EEMD denoising by a universal threshold formula as follows:
  (10) $$\sigma_{p}=median(|IMF_{p}|)/0.6745\;T_{p}=\sigma_{p}\sqrt{2\ln(N)}$$
  where $\sigma_{p}$ is the standard deviation of the noise, N is the length of $IMF_{p}$.

After determining the universal threshold $T_{p}$, the soft threshold function is selected to filter noise component coefficients in the high frequency IMFs

(11) $$IMF_{th}{}_{p}=\left\{ \begin{array}{l} \mathrm{sgn}(IMF_{p})(|IMF_{p}-T_{p}|),|IMF_{p}|\geq T_{p}\\ 0,\;\;\;\;\;\;\;\;\;\;\;\;\;\;\;\;\;\;\;\;\;\;\;\;\;\;\;\;\;\;\;\;\;\;\;\;\;\;\;\;\;\;|IMF_{p}|<T_{p} \end{array}\right.;$$

(6) Finally, EEMD denoising is given by:
  (12) $$X_{}^{i}{}_{dn}=\sum_{p=1}^{p_{1}}IMF_{th}{}_{p}+\sum_{p=p_{1}+1}^{P}IMF_{p}+r\;.$$

### 5.2. Sand Wave Online Detection

**Figure 3:**
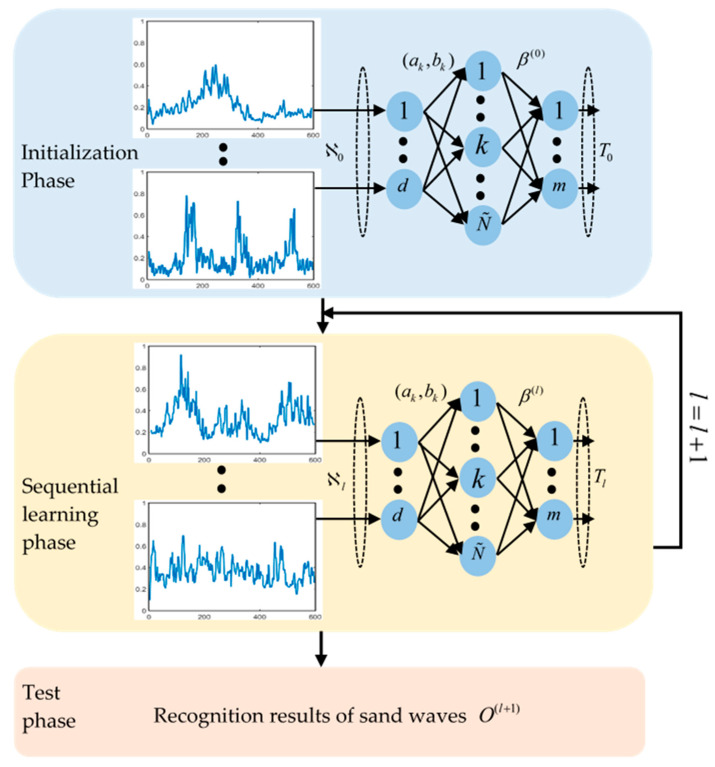
The flowchart of OS-ELM learning.

In this paper, we make an attempt to explore an online auto-detection technique for submarine sand waves through echo intensity, with the help of the OS-ELM learning framework. OS-ELM inherits the advantage of ELM, which can provide good generalization performance at an extreme learning speed, moreover, OS-ELM [39] has an online sequential learning capability that does not require retraining when new observations arrive, and especially adapts to rapid responses to AUVs dynamically. The reason why we emphasize the online computation is that we attempt to explore the data-driven mode that could perform sand wave detection during the voyage, rather than from postprocessing after sailing back. The data-driven scheme could provide the AUV system with the capacities to execute complex real-time processing and decision making, with a resulting increase in its autonomy, flexibility, and adaptability, multiplying their data collection efficiency. An AUV could hereby modify the path planning during the mission and approach regions with sand waves, which is conducive to reducing the time complexity of our survey and improving the quality and efficiency of sand wave inspection tasks. We progressively collect SSS imaging, extract the echo intensity, and then perform preprocessing such as blind zone removal, TVG correction, speed correction, EEMD denoising, and so forth and further try to establish single hidden layer feedforward neural networks (SLFNs) to derive the relevant mathematical criterion for the optimization problem of sand wave detection. The flowchart of OS-ELM learning is shown in Figure 3, with the following steps.

#### 5.2.1. Initialization Phase

(1) Input a small chunk of echo intensity sub-sequence ${ℵ}_{0}^{}={\left\{\left(X_{\bar{j}}^{},t_{\bar{j}}^{}\right)\right\}}_{\bar{j}=0}^{N_{0}}$. Set the number of hidden neurons as $\tilde{N}$, the training sample $X_{\bar{j}}^{}={\left[x_{\bar{j}1}^{},x_{\bar{j}2}^{},\cdots ,x_{\bar{j}d}^{}\right]}^{T}$, the number of $X_{\bar{j}}^{}$ in ${ℵ}_{0}^{}$ as $N_{0}$, the target output matrix as $T_{0}^{}={\left[t_{1}^{},\cdots ,t_{\bar{j}}^{},\cdots ,t_{N_{0}^{}}^{}\right]}_{1\times N_{0}^{}}^{T}$ and class label as $t_{\bar{j}}={\left[t_{\bar{j}1}^{},t_{\bar{j}2}^{}\cdots ,t_{\bar{j}m}^{}\right]}_{}^{T}$;
(2) Randomly assign the input weights $a_{k}^{}$ and bias $b_{k}^{}$. Set the output of the kth hidden node with respect to the input $X_{\bar{j}}$ as activation function $G(a_{k}^{},b_{k}^{},X_{\bar{j}}^{})=1/(1+e^{-(a_{k}^{}X_{\bar{j}}^{}+b_{k}^{})})$;
(3) Calculate the initial hidden layer output matrix $H_{0}^{}$:
  (13) $$H_{0}^{}=[\begin{array}{l} G(a_{1}^{},b_{1}^{},X_{1}^{})\cdots \\ \;\;\;\;\;\;\;\;\;\;\;\;\vdots \\ G(a_{1}^{},b_{1}^{},X_{N_{0}^{}}^{})\cdots \end{array}{\begin{array}{l} G(a_{\tilde{N}}^{},b_{\tilde{N}}^{},X_{1}^{})\\ \;\;\;\;\;\;\;\;\;\;\;\vdots \\ G(a_{\tilde{N}}^{},b_{\tilde{N}}^{},X_{N_{0}^{}}^{}) \end{array}]}_{N_{0}^{}\times \tilde{N}};$$
(4) Estimate the initial output weight $\beta^{\left(0\right)}$.

Calculate the intermediate variable $P_{0}^{}={\left(H_{0}^{}{}^{T}H_{0}^{}\right)}^{-1}$ and initial output weight $\beta^{\left(0\right)}=P_{0}^{}H_{0}^{}{}^{T}T_{0}^{}$ by the Moore–Penrose pseudo-inverse;

(5) Set l=0.

#### 5.2.2. Sequential Learning Phase

(6) Set the number of training data in the (l+1)th group as $N_{l+1}^{}=\sum_{p=0}^{l+1}N_{p}^{}-[(\sum_{p=0}^{l}N_{p}^{})+1]$. Input the (l+1)th echo intensity sub-sequence chunk ${ℵ}_{l+1}^{}={\left\{\left(X_{\bar{j}}^{},t_{\bar{j}}^{}\right)\right\}}_{\bar{j}=(\sum_{p=0}^{l}N_{p}^{})+1}^{\sum_{p=0}^{l+1}N_{p}^{}}$. Set the target output matrix of those $N_{l+1}^{}$ training data as $T_{l+1}^{}={\left[t_{(\sum_{p=0}^{l}N_{p}^{})+1}^{},\cdots ,t_{\sum_{p=0}^{l+1}N_{p}^{}}^{}\right]}^{T}$;
(7) Compute the partial hidden layer output matrix $H_{l+1}^{}$
  (14) $$H_{l+1}^{}=[\begin{array}{l} G(a_{1}^{},b_{1}^{},X_{(\sum_{p=0}^{l_{}}N_{p}^{})+1}^{})\cdots \\ \;\;\;\;\;\;\;\;\;\;\;\;\;\;\;\vdots \\ G(a_{1}^{},b_{1}^{},X_{\sum_{p=0}^{l+1}N_{p}^{}}^{})\cdots \end{array}{\begin{array}{l} G(a_{\tilde{N}},b_{\tilde{N}},X_{(\sum_{p=0}^{l}N_{p}^{})+1}^{})\\ \;\;\;\;\;\;\;\;\;\;\;\;\;\;\;\vdots \\ G(a_{\tilde{N}},b_{\tilde{N}},X_{\sum_{p=0}^{l+1}N_{p}^{}}^{}) \end{array}]}_{N_{l+1}^{}\times \tilde{N}};$$
(8) Calculate the output weight $\beta^{\left(l+1\right)}$,
  (15) $$P_{l+1}^{}=P_{l_{}}^{}-P_{l}^{}P_{l+1}^{}{}^{T}{\left(I+H_{l+1}^{}P_{l+1}^{}H_{l+1}^{}{}^{T}\right)}^{-1}H_{l+1}^{}P_{l_{}}^{}$$
  (16) $$\beta^{\left(l+1\right)}=\beta^{\left(l\right)}+P_{l+1}^{}H_{l+1}^{}{}^{T}(T_{l+1}^{}-H_{l+1}^{}\beta^{\left(l\right)})$$

Set l=l+1.

Go to Step 2.

#### 5.2.3. Test Phase

(9) Input test samples ${ℵ}_{l}^{*}=\{X_{(\sum_{p=0}^{l-1}N_{p}^{*})+1}^{*},\cdots ,X_{\bar{j}}^{*},\cdots ,X_{\sum_{p=0}^{l}N_{p}^{*}}^{*}\}$. Set the number of test samples ${ℵ}_{l}^{*}$ as $N_{l}^{*}=\sum_{p=0}^{l}N_{p}^{*}-[(\sum_{p=0}^{l-1}N_{p}^{*})+1]$;
(10) Predict class labels $O^{\left(l+1\right)}$
  (17) $$\begin{array}{l} O^{\left(l+1\right)}=H_{l+1}^{*}\beta^{\left(l+1\right)}\\ =[\begin{array}{l} G(a_{1}^{},b_{1}^{},X_{(\sum_{p=0}^{l}N_{p}^{*})+1}^{*})\cdots \\ \;\;\;\;\;\;\;\;\;\;\;\;\;\;\;\vdots \\ G(a_{1}^{},b_{1}^{},X_{\sum_{p=0}^{l+1}N_{p}^{*}}^{*})\;\;\;\;\;\;\cdots \end{array}{\begin{array}{l} G(a_{\tilde{N}},b_{\tilde{N}},X_{(\sum_{p=0}^{l}N_{p}^{*})+1}^{*})\\ \;\;\;\;\;\;\;\;\;\;\;\;\;\;\;\;\;\vdots \\ G(a_{\tilde{N}},b_{\tilde{N}},X_{\sum_{p=0}^{l+1}N_{p}^{*}}^{*}) \end{array}]}_{N_{l+1}^{*}\times \tilde{N}}\beta^{\left(l+1\right)} \end{array}$$

### 5.3. Morphological, Geometrical, Topological Characterization

#### 5.3.1. Morphological Template Making

**Figure 4:**
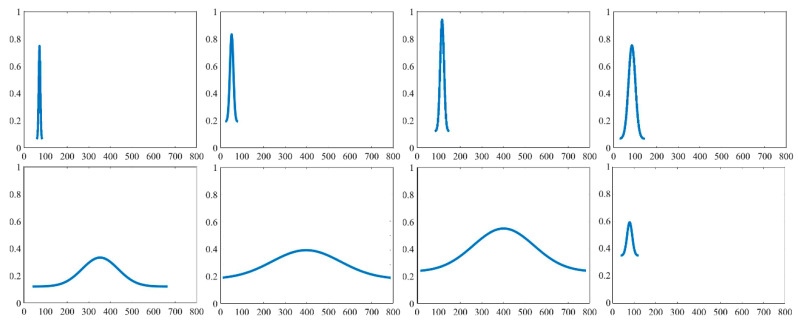
Example Gaussian-type templates.

Although the waveform of sand waves reflected from SSS imaging could exhibit unique and variable and sometimes rather complicated shapes, the majority forms repeatable patterns which can be grouped into a number of classes represented by typical morphological patterns, which may strongly depend on the morphological parameters such as the wave length, the wave height, and asymmetry index of submarine sand waves. We hereby denote a collection of Gaussian-type templates $T_{m}$ to simulate the fluctuation characteristics of sand waves:

(18) $$T_{m}=b_{t}+\exp\left(-{\left(x-\mu_{t}\right)}^{2}/2\sigma_{t}{}^{2}\right)/(\sigma_{t}\sqrt{2\pi })$$

where $\mu_{t}$ is the mathematical expectation of the Gaussian function, $\sigma_{t}$ represents the standard deviation of Gaussian function, and $b_{t}$ determines the starting point of the Gaussian function. The example Gaussian-type templates we have established for sand wave location and morphological extraction are shown in Figure 4, where $b_{t}$ is defined as $(0.1+0.02\times s_{d})$, $s_{d}\in [1,20]$ controls the moving stride of Gaussian-type template $T_{m}$ on the y-axis, $\mu_{t}$ ranges from $600\times u_{d}$, $u_{d}\in [0,15]$ controls the moving stride of Gaussian-type template $T_{m}$ on the x-axis, and $\sigma_{t}$ is chosen as $(1+0.2\times w_{d})$, $w_{d}\in [0,10]$ controls the dispersion degree of Gaussian-type templates $T_{m}$.

#### 5.3.2. Morphological Matching Criterion

We further establish one integrated template matching criterion for the morphological similarity evaluation in terms of cross-correlation r(X,T) and RMSE J(X,T), which aims at enhancing assessment in spatial similarity.

According to Taylor’s formula, $\ln(1+x)=x-x^{2}/2+x^{3}/3+\ldots +{\left(-1\right)}^{n}x^{n}/n$ and $\underset{x\to 0}{\lim}\ln(1+x)/x=1$, we constrain the similarity assessment by a logarithmic function, so as to construct the following template matching criterion for the echo intensity of sand waves:

(19) $$G_{t}=r(\chi_{j}^{i},T_{m})+\ln(1+1/J(\chi_{j}^{i},T_{m}))$$

where $\chi_{j}^{i}={\left[x_{j1}^{i},x_{j2}^{i},x_{jm}^{i}\cdots x_{jM}^{i}\right]}_{1\times M}$ is the portion of the jth sub-sequences in ith ping echo intensity used to match the template, $x_{jm}^{i}$ represents the mth echo intensity value. Additionally, the $T_{m}={\left[t_{1},t_{2},t_{m},\cdots t_{M}\right]}_{1\times M}$ is the Gaussian template, which not only considers the template matching between waveforms at the identical scales, but also the similarity measure in the context of geometric morphology. The logarithmic function is deployed to reduce the computational cost when the RMSE value is too small. The basic morphological descriptive parameters for sand waves will be hereby automatically quantified and recorded through the echo intensity during the template matching processes, including the wave height, wave length, and asymmetry index of sand waves, which would be probably helpful in further discussion on the sand wave migration rate and direction, the hydrodynamic analysis, and the stability of underwater engineering and even drive our AUV to sail along the optimal route planning to collect the specified type of sand wave more specifically.

#### 5.3.3. Zero-Crossing Rate

We further utilized the zero-crossing rate (ZCR) to examine the ratio of the sign changes of the waveform spectrum for sand waves, i.e., the number of times from the positive to negative or a reverse direction in a given period. Regardless of the scales and amplitudes submarine sand waves may exhibit, from small-scale megaripples to large-scale sand banks, they do have the periodic morphological characteristics reflected from the waveform of echo intensity. On the contrary, the echo waveform without sand waves tends to be distributed less orderly with random noises of high-frequency. The waveform in the sand wave region could be seen as the amplitude modulation process, with the random noise of high-frequency as the carrier and the sand wave morphology of relatively low-frequency as the modulating signal. The attribute of waveform ZCR is not related to the amplitude itself, with a strong anti-interference ability. As the waveform spectrum maintains more periodicity, ZCR values calculated for a certain period approach a constant value. Intuitively, the greater number of zero crossing of echo intensity in SSS imaging is, the more rapid changes and, accordingly, a high frequency spectrum it contains. Thus, ZCR could provide indirect clues about the frequency contents for sand waves. We make an attempt to identify the topology in submarine sand waves by counting the number of times they cross zero in the echo intensity and quantify the periodic variation. An extension of ZCR could be taken as a discriminative feature descriptor of the frequency content for low sampling frequency waveforms employing the existence of underlying information related to sand waves detection and morphological characteristics. The waveform with sand waves bear a low frequency range of ZCR which can be distinguished from noise and eliminate the noise and vibration of waveforms in regions without sand waves. ZCR could be denoted as:

(20) $$Z^{’}=\frac{1}{2}\sum_{v=2}^{V}\left|\mathrm{sgn}[x_{v}^{i}-{\bar{X}}_{}^{i}]-\mathrm{sgn}[x_{\left(v-1\right)}^{i}-{\bar{X}}_{}^{i}]\right|/\left(V-1\right)$$

where $x_{v}^{i}$ and $x_{\left(v-1\right)}^{i}$ are the vth and (v−1)th echo intensity of the sonar signal $X_{}^{i}$, ${\bar{X}}_{}^{i}$ is the mean of $X_{}^{i}$ and V is the length of $X_{}^{i}$. sgn[⋅] is a symbolic function as follows:

(21) $$\mathrm{sgn}[x_{v}^{i}]=\left\{ \begin{array}{l} 1,x_{v}^{i}>0\\ -1,x_{v}^{i}<0 \end{array}\right..$$

## 6. Simulation Experiment and Results Analysis

**Figure 5:**
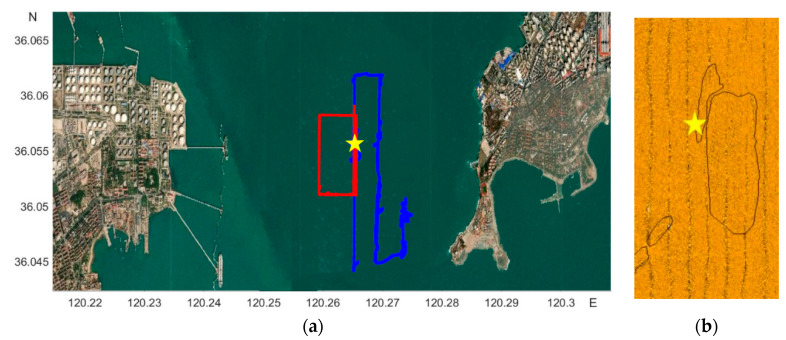
AUV trajectories and the manually labeled sand wave region. (**a**) AUV trajectories. (**b**) The manually labeled sand wave region.

In our simulation experiment, we repeatedly deployed our AUV system around Jiaozhou Bay, Qingdao, China on 6 December 2019 and 13 January 2021 for sand wave inspection, respectively starting at 36°02′38″ N, 120°16′1″ E and 36°03′27″ N, 120°15′48″ E, and ending at 36°03′43″ N, 120°16′26″ E and 36°03′0″ N, 120°16′2″ E, by the high-resolution SSS of 150 kHz frequency, 200 m operating ranges per side, 1.25 cm across track resolution, 0.6° horizontal beam width, 40° vertical beam width, as is shown in Figure 5, with the first and second AUV trajectories respectively represented in blue and red on the left, and the manually labeled sand wave region on the right. The SSS imaging log with two AUV deployments is listed in Table 1.

**Figure 6:**
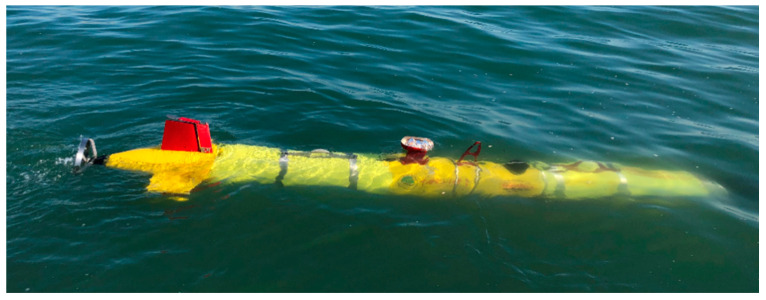
AUV deployment.

**Figure 7:**
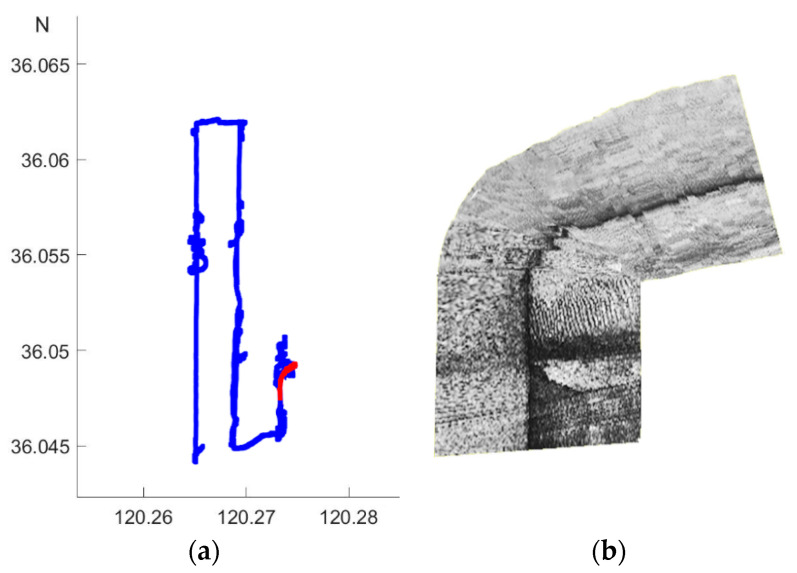
AUV path and SSS mosaicking. (**a**) AUV path. (**b**) SSS mosaicking.

The diameter, length, and weight of our AUV system is respectively 324 mm, 4 m and 280 kg, as is shown in Figure 6. A high-performance Industrial Personal Computer PC104, together with the NVIDIA Jetson TX2 platform established the distributed control architecture with the super computation unit, with the sensors and measurement instrument on board, including lcocean Shark-S450D side scan sonar, u-blox NEO-M8T GPS, Linkquest Navquest micro 600 doppler velocity log (DVL), Beidou era BD-FGI920 inertial navigation system (INS), Valeport miniIPS intelligent pressure sensor, Evologics S2C acoustic communication, and so forth. With the help of the positioning and navigation, we made SSS mosaicking during postprocessing, as shown in Figure 7, where on the left, the AUV path of the first time is in blue, with the route of the stitched image in red as an example, and the corresponding mosaicking result on the right.

**Figure 8:**
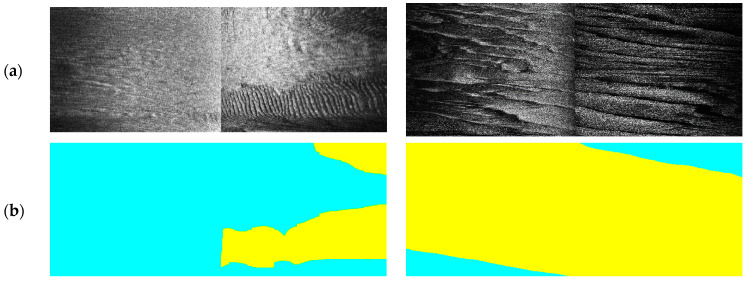
Example SSS annotation. (**a**) Example SSS imaging. (**b**) Labeling.

At the beginning, we utilized the manually labeled sand wave annotation in historic surveys by research vessel with the high resolution SSS on board for the standard references. During the process of SSS imaging acquisition by AUV, we further accumulated and refined the sand wave annotation as our ground truth. Figure 8 shows the example SSS imaging collected and the corresponding labeling, with sand waves in yellow and non sand waves in blue, respectively.

**Figure 9:**
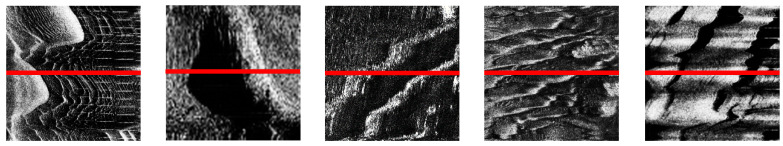
Example echo intensity sub-sequence extraction from raw SSS imaging.

We retrieved the echo intensity in each ping and then divided these into overlapping sub-sequences with the basic length of 600. Considering the operating range of 200 m per side, we made an initial estimation to cover the seabed 25 m for each sub-sequence. In total, 327,280 echo intensity sub-sequences were chosen for OS-ELM learning and statistical analysis, with 156,306 samples labeled in the category of sand waves, and 170,974 samples in the class without sand waves. For each class, we set aside one fifth for test and verification, respectively, and the rest for training. Figure 9 lists the example echo intensity sub-sequence extraction process from raw SSS imaging, with the centered red line indicating the extraction localization of echo intensity sub-sequence from raw SSS imaging patches.

**Figure 10:**
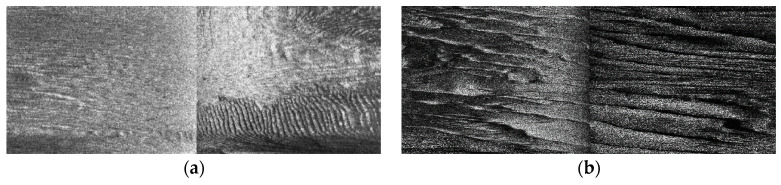
TVG correction for example SSS imaging. (**a**) The corrected example SSS imaging 1. (**b**) The corrected example SSS imaging 2.

**Figure 11:**
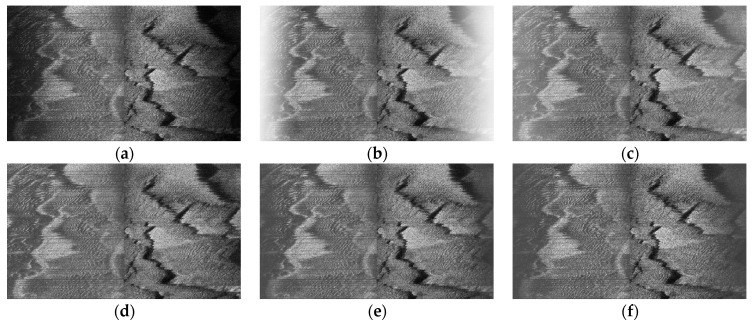
TVG correction performance comparison. (**a**) Raw data. (**b**) A=30, B=20. (**c**) A=15, B=10. (**d**) A=5, B=3.1. (**e**) A=4, B=3. (**f**) A=2, B=1.

Considering the typically uneven characteristics on across-track in SSS imaging, we made TVG correction against these effects, with the empirical correction index *A* = 5 and empirical correction bias B=3.1, and the corrected example SSS imaging of Figure 8a has been shown in Figure 10. In this context, we carried out a series of TVG correction experiments to analyze the selection of the relatively optimal parameters over our study area, with the combination of typical values A=30, B=20; A=15, B=10; A=5, B=3.1, A=4, B=3; A=2, B=1, and so forth. Some of the TVG correction examples are shown in Figure 11, where both overcorrection and undercorrection occurred for some pairs of parameters, resulting in either secondary gray distortion or edge texture weakening. When we chose A=5, B=3.1, the radiometric distortion could be well eliminated with TVG correction, resulting in a relatively realistic reflection of the seafloor. Instead of only generating enhanced contrast processing, TVG correction provided the acoustic energy compensation with the function of echo attenuation and propagation distance.

**Figure 12:**
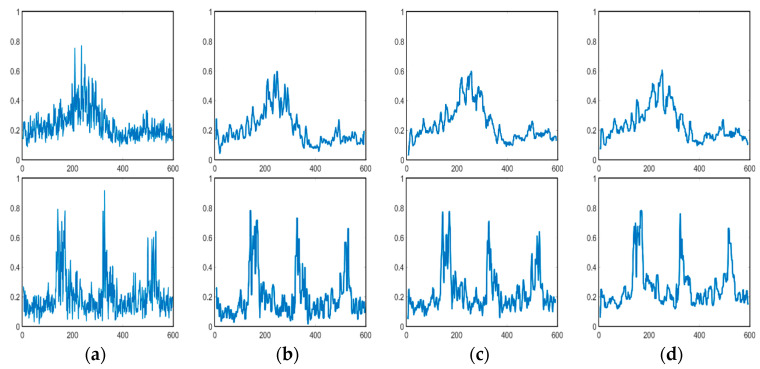
Example sand wave profile waveform and denoising. (**a**) Raw echo intensity sub-sequence. (**b**) Median filtering. (**c**) Gaussian filtering. (**d**) EEMD denoising.

We verified the validity of EEMD denoising to highlight the morphology of sand wave profile waveform with higher smoothing capability as well as edge preservation, in comparison to a few classical filters, such as the median filter and Gaussian filter, as is shown in Figure 12, with the common sliding window size $l_{f}=3$ and the standard deviation of Gaussian filter $\sigma_{gf}=0.8$.

**Figure 13:**
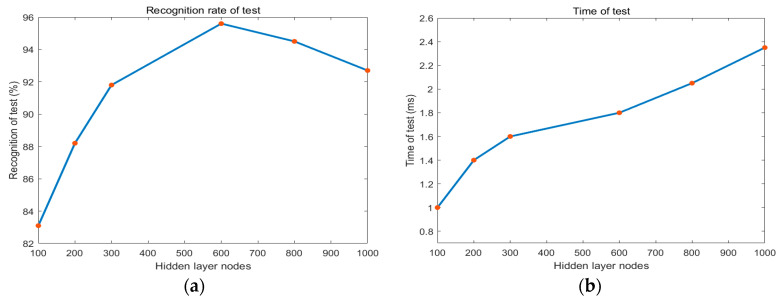
Sand wave detection rate and time regarding to the number of hidden layer nodes in OS-ELM. (**a**) Test recognition rate. (**b**) Test time.

We further established OS-ELM architecture and fed into training samples for sand wave detection. Regarding the model selection, the average detection accuracy has been taken to assess across the configuration of hyper parameters. The number of hidden layer nodes has been considered to avoid the learning contingency and the corresponding test time has also been recorded, as is shown in Figure 13. It has been demonstrated from the experimental results that the sand wave detection rate initially increased when the relatively low number of hidden layer nodes rose until it reached 600, with the sand wave detection rate of 95.61% and the test time of 0.0018 s on average, and then the tendency of the detection rate remained or decreased after the certain value.

**Figure 14:**
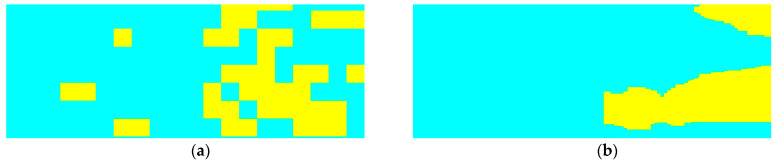
Sand wave detection for example SSS imaging. (**a**) Basic MobileNet with SSS imaging. (**b**) OS-ELM with echo intensity.

We also employed the basic MobileNet architecture [61] to perform sand wave detection with SSS imaging in the context of one light-weight deep learning framework. The sand wave detection results of example SSS imaging in Figure 11a have been compared in Figure 14, where the left is from the basic MobileNet by SSS imaging, and the right is from OS-ELM by echo intensity, with sand waves in yellow and non sand waves in cyan, respectively. It can be seen from our experiment results that MobileNet aimed to the sand wave detection in a large range, while our proposed scheme could concentrate more on the details of the sand wave profile distribution in every ping, especially showing superior performances for those sand waves in smaller sizes, e.g., megaripples.

We have also evaluated the sand wave detection performances of our proposed approach comprehensively in terms of the detection Accuracy, False Negative Rate (FNR), False Positive Rate (FPR), and F1-Score, and the evaluation metrics are defined as:

(22) $$\begin{array}{c} FPR=FP/(FP+TN)\\ FNR=FN/(TP+FN)\\ Pression=TP/(TP+FP)\\ Recall=TP/(TP+FN)\\ Accuracy=\left(TP+TN\right)/(TP+FP+TN+FN)\\ F1-Score=2\times \left[Precission\times Recall/\left(Precission+Recall\right)\right] \end{array}$$

where True Positive (*TP*) refers to the set of echo intensity sub-sequence which falls into the sand wave class and is correctly classified. False negative (*FN*) refers to the set of echo intensity sub-sequence which falls into the sand wave class but is misclassified as the background. True negative (*TN*) refers to the set of echo intensity sub-sequence which falls into the background and is correctly classified. False Positive (*FP*) refers to the set of echo intensity sub-sequence which falls into the background class but is misclassified as the sand waves. The sand wave detection performances of AUV inspection in December 2019 and January 2021 are listed in Table 2, Table 3 and Table 4. It is revealed in our experiment that the sand wave detection rate with echo intensity could reach 95.61% averagely, and FNR 4.36%, FPR 4.82%, F1-Score 95.32%, with the average test time 0.0018 s and the total training time 5.7168 s for sea trials, comparable to MobileNet, but at a much higher speed and low computational cost, showing its superiority particularly for sand waves at smaller scales, such as megaripples.

Generally speaking, OS-ELM architecture for sand wave detection shows its adaptivity, but most of the difficulty still tends to come from the availability of qualified echo intensity waveform from SSS profile when deploying our AUV system. We also discussed the generalization capacity of our OS-ELM learning procedure for different spots and seasons with different states of AUV. We performed the OS-ELM learning framework in the training dataset at different spots, e.g., the study area around the South China Sea, and achieved the comparable average detection rate of 90.57%. We also carried out the simulation experiment in the study area of Jiaozhou Bay with the training samples respectively collected from different seasons, i.e., in December 2019, June 2020, and January 2021, and the sand wave detection rates reached 95.42%, 94.57%, and 95.67%. Although the migration and morphology of sand waves could be related to meteorological conditions, it has been demonstrated that by now the training procedure still behaved relatively stably, with a relatively reasonable impact on sand wave detection. In practice, we usually deploy our AUV system for a sand wave detection task under the level 3 sea state and collect SSS data in the relatively stable operation stage of AUV. We gather AUV motions of surge, sway, heave position, and angular rotations of roll, pitch, and yaw angle and also help with the compensation and correction of the position offset of the echo intensity in the along- and across-track directions to the navigation trajectory. Therefore, the learning procedure have been designed to perform relatively adaptively for some general situation, in sand wave detection applications.

**Figure 15:**
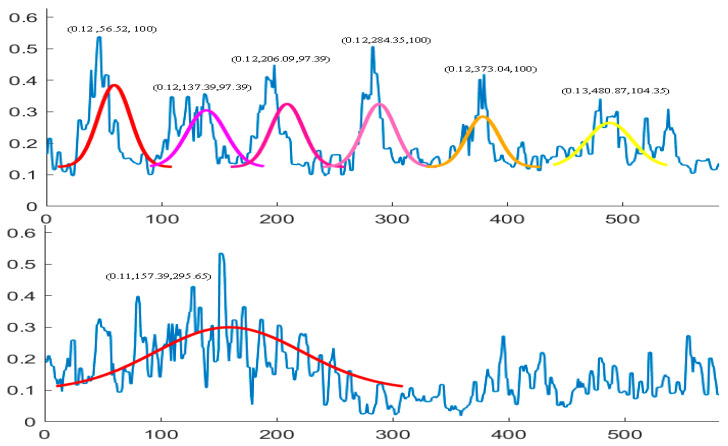

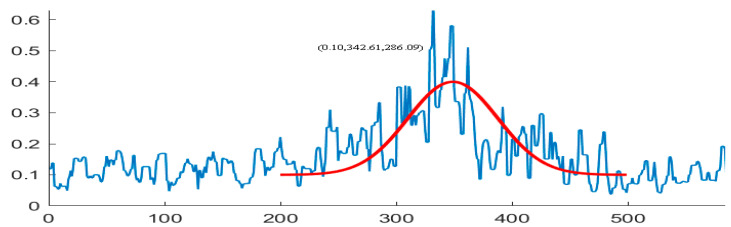
Example sand wave profile waveform matching.

We not only examined the existence of sand waves in the study area, but also focused on investigating what type of morphological properties the sand waves would probably possess. Therefore, we also established a variety of deformable slide Gaussian templates to match the sand wave profile waveform with the similarity metric. Some example Gaussian template matching process has been shown in Figure 15, with an average calculation speed of 0.53 s for each echo intensity sub-sequence in locating all the matching sand waves.

**Figure 16:**
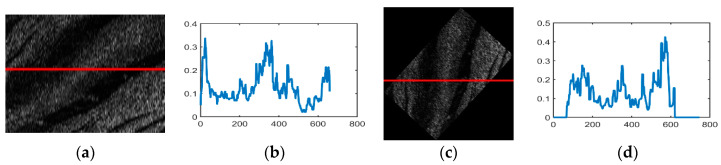
Geometric correction example. (**a**) Raw SSS imaging. (**b**) Raw echo intensity values. (**c**) SSS imaging after geometric correction. (**d**) Echo intensity values after geometric correction.

When the AUV system sails through the sand wave region, it is possibly not aligned perfectly with ridge lines. If there exists an angle between the direction vector of AUV with the front wave axis, on the basis of the edge detection in both horizontal and vertical directions, we could try to first estimate the horizontal tilt angle α and vertical tilt angle β by Radon transform, and then make use of shear transformation for the geometric correction of the entire SSS image before morphological template matching as follows,

(23) $$\left[\begin{array}{c} {\tilde{x}}^{\prime }\\ {\tilde{y}}^{\prime } \end{array}\right]=\left[\begin{array}{cc} 1 & -\sin(\alpha \times \pi /180)\\ -\sin(\beta \times \pi /180) & 1 \end{array}\right]\times \left[\begin{array}{l} \tilde{x}\\ \tilde{y} \end{array}\right]$$

where $\tilde{x}$ and $\tilde{y}$ are the abscissa and ordinate of the image pixels in the original SSS imaging,${\tilde{x}}^{\prime }$ and $\tilde{y}\prime$ are the abscissa and ordinate of transformed image pixels in the newly generated SSS imaging. Figure 16 shows one rotation transformation example to SSS imaging with sand wave regions, before applying morphological Gaussian templates.

## 7. Discussion

From the perspective of echo intensity measurement in SSS imaging, sand waves appear highly irregular in size, shape, and spacing. In order to investigate the variability of geometrical properties in sand waves, it is of great importance to identify the locations and main directions of the crests and troughs for sand waves in their individual SSS profiles during the hydrodynamic and sediment dynamic processes.

We were hereby inspired to make the upper and lower envelopes for sand wave profile to compose the reconstructed boundaries within which the maxima and minima signals in echo intensity are contained in the following steps: (1) identify all the local extrema; (2) interpolate between extrema by a cubic spline. Since the geomorphology of sand waves would produce vibration signals of low frequency in echo intensity, the envelope demodulation process reflects the amplitude changes and direction of the sand wave signals with the extreme of the waveform, detect the vibration with low signal-to-noise ratio from massive noises by filtering, and further help determine sand waves types.

**Figure 17:**
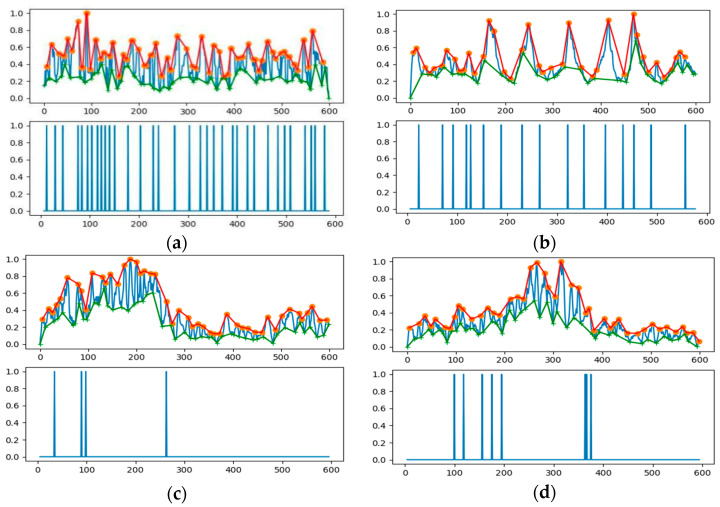
Zero-crossing spectrum. (**a**) Without sand wave. (**b**–**d**) With sand waves.

**Figure 18:**
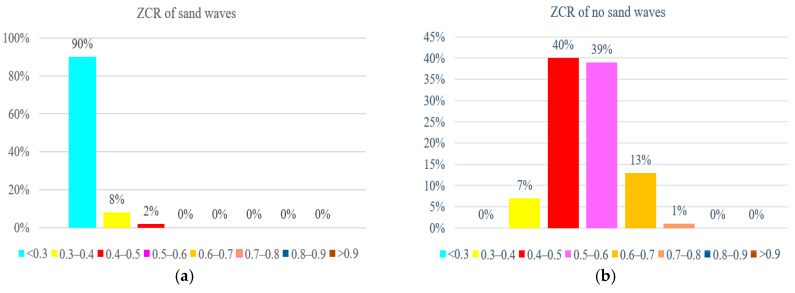
ZCR statistics. (**a**) With sand waves. (**b**) Without sand waves.

We further examined the zero-crossing rate spectrum for the example envelope demodulation, as is shown in Figure 17, where the crests and troughs could also be recognized by mapping between zero upcrossings and zero downcrossings in the bedform SSS profile with stability. We also made a statistical comparison of ZCR extracted from the echo intensity with or without sand waves in our study area when we deployed our AUV to Jiaozhou Bay for SSS imaging collection during the sea trial in January 2021, as is shown in Figure 18, and found that ZCR remained between 0.3–0.4 in the regions with sand waves and ranged from 0.4–0.6 for the regions without sand waves, with an average calculation speed of 0.56 ms for each echo intensity sub-sequence. It has been estimated from the experimental result that, compared to the regions with sand waves, those regions without sand waves might most probably bear the relatively large zero-crossing rate percentage and the denser zero-crossing spectrum in higher frequency, implying that the echo intensity with sand waves is of stronger regularity in a relatively low frequency, while the ZCR without sand waves tends to be distributed more disorderly and varies more frequently, so that we could further exclude the influence of noise interference in SSS imaging. To employ ZCR might possibly provide one of the distinguishing attributes in analyzing morphological properties for sand waves, which could also help reduce dimension, remove redundancy, improve the computational cost.

**Figure 19:**
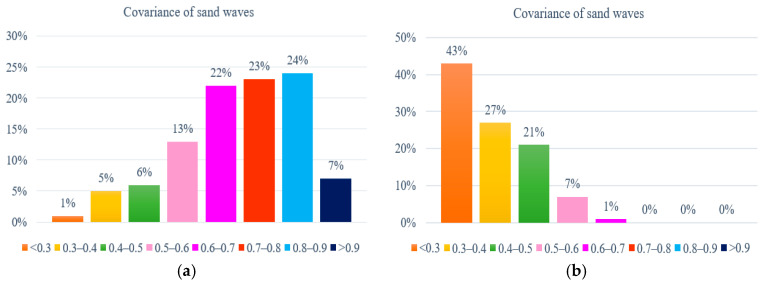
Cross-correlation statistics. (**a**) With sand wave. (**b**)Without sand wave.

Furthermore, we established cross-correlation coefficients to explore a broad class of relationships among upper and lower envelope demodulation and evaluated the strength via the covariance matrix, either for a sample of echo intensity or a population within the whole research area. Figure 19 refers to the statistics of cross-correlation coefficients between the upper and lower envelopes in SSS profiles on both sand wave and non sand wave regions for our study area, with an average calculation speed of 0.12 s for each echo intensity sub-sequence. It has been demonstrated that for the local regions with the sand waves, the upper and lower envelopes co-moved with each other synchronously, and the cross-correlation coefficient was relatively large, implying that the amplitude rises and falls in sand waves could roughly reflect the fluctuation of submarine topography through the nearly periodic variation of echo intensity in SSS profiles. It has been calculated that when the cross-correlation coefficient was greater than 0.75, the possibility of the existence of sand waves approached 90% in terms of the detection precision. In the regions without sand waves, the cross-correlation coefficients remained relatively small, exhibiting the disaccordance and inconsistence in their distribution, which indicates the randomness and independence with more disorderly variation.

We further discussed how to refine the specific morphological parameters by means of echo intensity in SSS profile, including wave length, wave height, asymmetric index. The hydrodynamic instability generates coherent flow structures and contribute to the formation of sand waves. The wave length and wave height are the most intuitive reflections of sand wave morphology. Let the span of a wave be the wave length $S_{L}$, which could be denoted as:

(24) $$S_{L}=\frac{S_{L}^{*}}{R_{H}^{*}}\times R_{H}$$

where $S_{L}^{*}$ is the length of echo intensity sub-sequence in SSS imaging, $R_{H}^{*}$ represents the maximum number of echo intensity per side in SSS imaging, $R_{H}$ stands for the real distance that SSS projects acoustic waves onto the seafloor on each side, which could be derived by the spatial relationship between the operating range per side and AUV height above the seabed.

**Figure 20:**
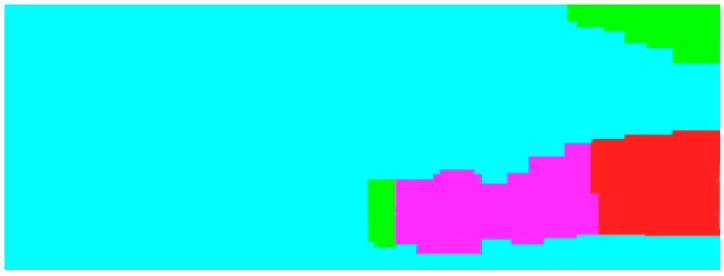
Wave length estimation distribution for sand waves.

We calculated the statistics for the wave length estimation as above. Figure 20 shows the wave length calculation for the example SSS imaging in Figure 10a, and we inferred that the wave length around that region primarily ranged between 10 m and 25 m, responsible for 78% of all types of sand waves, which shows a significant consistency with the historic surveys executed by research vessels with a high resolution SSS on board. More precisely, in review of the whole region after sand wave detection, the sand waves at the wave length of 10–15 m in rose color accounted for 46% of all sand waves, the sand waves at the wave length of 15–25 m in red color almost reached to 32%, while the sand waves at the wave length less than 10 m in green color constituted 22%. However, the proposed scheme is more applicable and beneficial to those morphological parameter calculations for sand waves when the wave ridge lines in survey is parallel to AUV path planning.

**Figure 21:**
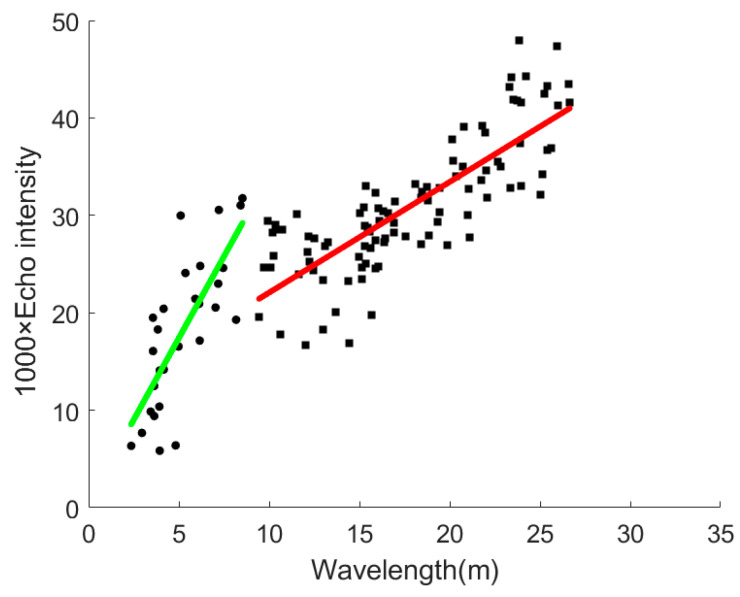
Morphological relationship of wave length estimation versus local maxima echo intensity for sand waves.

We also analyzed the statistical morphological relationships between the wave length estimation in every single waveform and the corresponding local maxima echo intensity value among the local minima for sand waves, as is shown in Figure 21. It could be seen from the scatter plot that the sand waves in that region could be further roughly classified into two types according to their relationship distribution, i.e., the shorter wave lengths within 10 m with smaller maxima echo intensity less than 0.025, and the longer wave lengths between 10–30 m with the larger maxima echo intensity around 0.032, which could be characterized and approximated respectively by two linear functions with the slope 3.35, 1.14 and the intercept 0.64, 10.71, regarding 1000 times the echo intensity versus the wave length.

Although the wave height of sand waves is unable to be retrieved from SSS imaging accurately, it has been reported in previous studies [3,62,63] that the wave height of sand waves exhibits the tendency to increase with the increasing wave length, which indicated that the two morphological descriptive indices of sand waves potentially possessed significant positive correlation with each other under certain conditions.

Flemming [63] statistically investigated 1491 sand waves all over the world and proposed the an equilibrium geometrical relationship between a wave height $S_{H}$ and the wave length $S_{L}$ as follows,

(25) $$S_{H}=0.0677{\left(S_{L}\right)}^{0.8098}.$$

Flemming [63] also put forward an upper limit $S_{H\max}^{*}$ for the wave height by:

(26) $$S_{H\max}^{*}=0.16{\left(S_{L}\right)}^{0.84}.$$

In a different equilibrium geometrical relationship between the wave height $S_{H}$ and the wave length $S_{L}$ proposed by Franzetti et al. [62], about 500 sand waves in the continental shelf of western Brittany were identified and measured as follows,

(27) $$S_{H}=0.0139{\left(S_{L}\right)}^{1.164}.$$

Notwithstanding that a large number of sand waves in various formation environments have been considered, there are still examples not well described by the above Flemming’s empirical relationships; e.g., Van Landeghem et al. [64,65] have observed a very large dune with a length of 435 m and a height of about 35.5 m at a water depth of 90 m in the Irish Sea which had a height larger than Flemming’s global mean regression would predict. Generally, Flemming’s wave height prediction could be regarded as one of the currently most authoritative and commonly used formulas for the two morphological descriptive indices of sand waves globally. According to the wave length estimation results in our study region, we applied the empirical relationship in Flemming’s formula and deduced the possible wave height estimation around 0.77 m and the upper limit about 1.98 m for SSS imaging in the study region. To be specific, the wave height and upper limit within the wave length 10 m were estimated to be 0.32 m and 0.82 m, while for the wave length between 10–30 m, 0.86 m and 2.23 m, which showed good agreement with the previous research on the morphological characteristics of submarine sand waves around our study region in Jiaozhou Bay [66,67].

**Figure 22:**
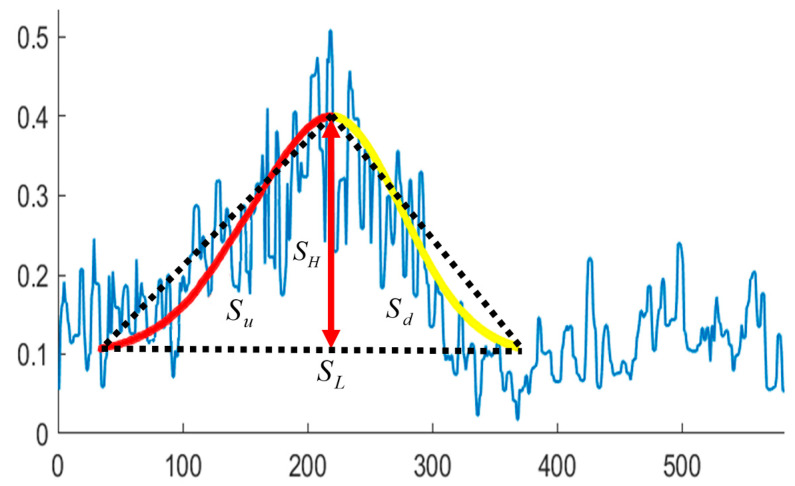
Asymmetric index definition.

The internal structure of the sand waves apparently depends on the strength and degree of asymmetry of the governing currents, and the relatively asymmetrical currents would shape sand waves with one side too steep. The bigger the difference between the upstream and downstream slope projection in the sand wave is, the bigger the flow velocity difference will be reflected, which is much closer to the one-directional flow, and the activity of sand waves would rise. Conversely, the activity of sand waves becomes weak with better stability. We hereby tried to calculate the values of the upstream and downstream slope projection in echo intensity and explore the asymmetric index of sand waves, as is shown in Figure 22. The ratio between the upstream slope projection $S_{u}$ and the downstream slope projection $S_{d}$ be one asymmetric index R,

(28) $$R=S_{u}/S_{d}$$

**Figure 23:**
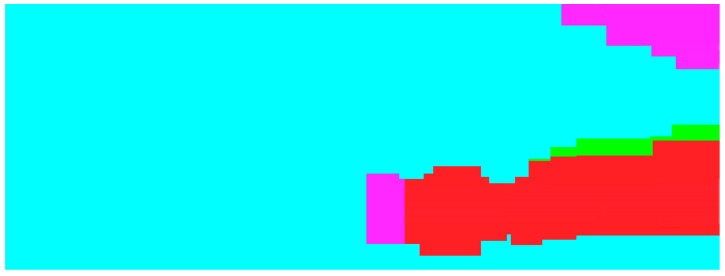
Asymmetrical index estimation distribution for sand waves.

The asymmetrical index estimation for sand waves in the above study region is shown in Figure 23. It has been demonstrated from our experimental results that those sand waves with the asymmetrical index R<0.5 in red color were calculated to be more likely to incline westward, the sand waves with the asymmetrical index 0.5<R<1.5 in rose color look to be relatively stable and balanced between the upstream and downstream slope projection from the echo intensity with the relatively weak activity in movement, and the sand waves with the asymmetrical index R>1.5 in green color were inferred to possibly lean eastward, respectively accounting for 72%, 22%, and 6% in the study region. As a whole, from the perspectives of morphology, the study region of sand waves would most probably be influenced by the westward tidal current compared to the eastward ones, implying the direction of hydrodynamic forces that represent the dominant ones in driving the sand wave movements, with a slightly higher strength compared to the other directions.

**Figure 24:**
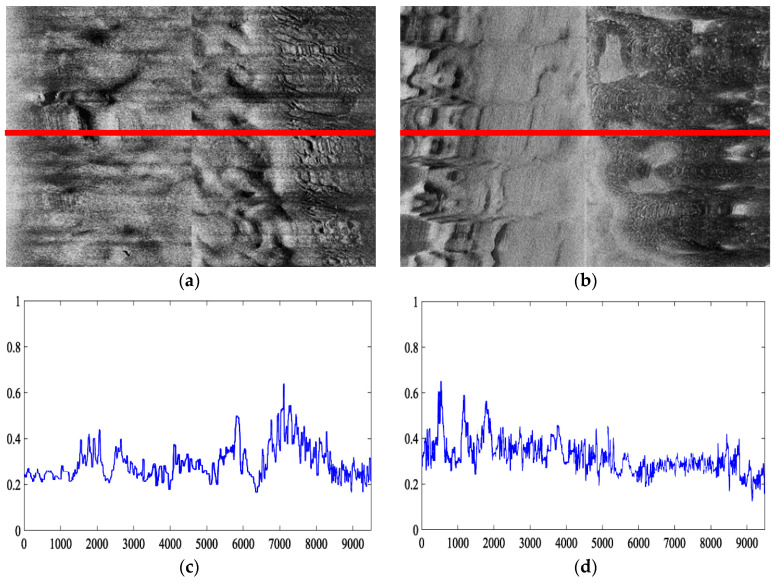
Example echo intensity in SSS imaging at the intersection of two AUV trajectories within the duration of more than one year. (**a**) SSS imaging in December 2019. (**b**) SSS imaging in January 2021. (**c**) Echo intensity curve regarding the ping in red on SSS imaging (**a**). (**d**) Echo intensity curve regarding the ping in red on SSS imaging (**b**).

It is generally accepted that the lee-stoss asymmetry of sand waves is a sign for sand wave migration in the direction of the steeper slope [68]. We have therefore deployed our AUV twice around the same study region in December 2019 and January 2021 and collected SSS imaging within the duration of more than one year to verify the estimated direction of sand wave migration. Regarding the intersection of AUV trajectories of both sea trials in the yellow star, the comparison of echo intensity in SSS imaging is illustrated in Figure 24, with the left collected in December 2019 and the right collected in January 2021, and the echo intensity curves refer to the ping in the red line located on SSS imaging. It has been demonstrated from SSS profiles that compared to the echo intensity curves before and after one year, there is indeed exhibited a westward migration tendency of the sand waves, with the asymmetry index less than 0.5, and the steep slopes were assumed to shape the westward. It has been reported that the tidal current in Jiaozhou Bay could be characterized as a regular semidiurnal one, towards the westward direction at the flood tide and to the eastward direction at the ebb tide, where the current at the flood tide has been supposed to be generally stronger than the one at the ebb tide [69]. Li et al. stated that there is a similar type of hydrodynamic environment around Jiaozhou Bay and demonstrated the impact in the tidal flow and tidal flux with the cross-bay bridge built [69]. Bian et al. [67] studied the distribution, morphology, and migration of submarine sand waves around Jiaozhou Bay by means of the multibeam bathymetric data, SSS data, and seismic profiles and found that the sand waves, mainly exhibiting straight crest type and crescentic type, moved westward under the control of the tidal current. Our findings through morphological parameters with echo intensity in SSS imaging agree with the previous field surveys and proved to some extent the effectiveness of the proposed scheme.

## 8. Conclusions

Submarine sand waves exhibit ubiquitous geomorphology at a variety of scales, including sand banks, sand waves, and megaripples, highly reflecting the coupling variation of multivariate marine environmental factors, such as the tidal characteristics, sediment grain sizes, related residual currents, and so forth. From the morphological and topological perspectives, submarine sand waves declare a uniform wavelength at equilibrium and develop defects while adjusting to variation. In this paper, we quantitatively explore the formation and migration cues in sand waves on topology, morphology, and evolution from high-resolution mapping via a side scan sonar (SSS) mounted in an Autonomous Underwater Vehicle (AUV), in favor of online sequential Extreme Learning Machine (OS-ELM). We first make an attempt to utilize echo intensities directly derived from SSS to help accelerate online detection and localization for sand waves, with the help of time varying gain (TVG) correction, speed correction, ensemble empirical mode decomposition (EEMD) denoising, and so on to compensate for the frequent motion variation on the AUV in synthesizing consecutive uniform pings of SSS imaging over time. We then denote a collection of Gaussian-type morphological templates to capture the fluctuation characteristics of sand waves and establish one integrated matching criterion for similarity assessment in terms of cross-correlation and RMSE. We statistically investigate the envelope demodulation, zero-crossing rate (ZCR) spectrum, and cross-correlation coefficients to examine the high variability of sand waves regarding their size, shape, and spacing and further potentially estimate the specific morphological parameters, including wave length, wave height, and asymmetric index, by means of echo intensity in the SSS profile. In our simulation experiment, we repeatedly deployed our AUV system around Jiaozhou Bay, Qingdao, China in 2019 and 2021, within the duration of more than one year, equipped with SSS of 150 kHz frequency, 200 m operating ranges per side, 1.25 cm across track resolution, 0.6° horizontal beam width, 40° vertical beam width, as well as a high-performance PC and GPU for control and online computation, DVL, INS, digital compass, and depth gauge for navigation. We established OS-ELM architecture, retrieved 327280 overlapping echo intensity sub-sequences, fed into 196368 training samples, and comprehensively evaluated the detection accuracy, False Negative Rate (FNR), False Positive Rate (FPR), and F1-Score with the sand wave annotation in historic surveys by research vessel and the increased manually labeling for the standard references. The model selection was elaborated by assessing the detection performances across the configuration of hyper parameters to determine the number of hidden layer nodes. It is revealed in our experiment that the sand wave detection rate with echo intensity could reach 95.61% averagely, and FNR 4.36%, FPR 4.82%, F1-Score 95.32%, with the average test time of 0.0018 s for sea trials, which is comparable to the image-based detection performances in the light-weight deep learning framework, e.g., MobileNet, but at a much higher speed and low computational cost, showing its superiority particularly for sand waves at smaller scales, such as megaripples. The statistics of ZCR and the cross-correlation coefficient between envelope demodulation illustrated differences in sand wave and non sand wave regions, implying the regularity, periodicity, and synchronousness in the low-frequency waveform of sand waves. We inferred that the wave length around that region primarily ranged between 10 m and 25 m, responsible for 78% of all types of sand waves, characterized and approximated respectively by linear functions with the slope 3.35, 1.14 and the intercept 0.64, 10.71, and the sand waves in the study would most probably exhibit the influences of the westward currents compared to the eastward ones, implying the possible dominant direction of hydrodynamics in driving the sand wave migration, which shows a strong consistency with previous studies. The proposed scheme permits to delicately explore the submarine geomorphology of sand wave variation, allowing the morphological imagery at a higher resolution and online computation strategies, greatly breaking through the restrictions of field survey with more flexibility and adaptivity, and symmetrically integrating the geometrical, topological, and morphological evidences yielded from echo intensity in SSS imaging, which would help provide opportunities for identifying the underlying dominantly correlative factors in nature and potentially suggest the leading roles in the responses of sand waves during their formation and migration.

## Figures and Tables

**Table 1:** SSS imaging with AUV deployment in Jiaozhou Bay.

Year	Name	Start and End Time	Duration	Ping Number
2019	Jiaozhou Bay 1	10:31:55–12:44:00	2:12:05	21868
2021	Jiaozhou Bay 2	14:00:23–16:32:52	2:32:29	25181

**Table 2:** Sand wave detection performance on Jiaozhou Bay 1.

Jiaozhou Bay 1	FNR (%)	FPR (%)	Accuracy (%)	F1-Score (%)	Training Time (s)	Testing Time (s)
MobileNet V3	4.93	6.37	94.69	94.07	9013.1247	0.0201
Ours	4.41	5.07	95.42	95.29	2.4765	0.0013

**Table 3:** Sand wave detection performance on Jiaozhou Bay 2.

Jiaozhou Bay 2	FNR (%)	FPR (%)	Accuracy (%)	F1-Score (%)	Training Time (s)	Testing Time (s)
MobileNet V3	4.65	5.49	95.32	95.27	10802.6745	0.0247
Ours	4.28	4.73	95.67	95.38	3.2179	0.0021

**Table 4:** Sand wave detection performance on Jiaozhou Bay 1&2.

Jiaozhou Bay 1 & 2	FNR (%)	FPR (%)	Accuracy (%)	F1-Score (%)	Training Time (s)	Testing Time (s)
MobileNet V3	4.73	6.25	94.87	94.47	18704.4657	0.0239
Ours	4.36	4.82	95.61	95.32	5.7168	0.0018

## Data Availability

Data are available from the author upon reasonable request. The data are not publicly available right now due to project privacy.

## Appendix A

1. Each Intrinsic Mode Function (IMF) of the *i*th ping echo intensity $X^{i}$ is defined as a function that must satisfy the following conditions,
  (1) In the whole data set, the number of extrema and the number of zero-crossings must be equal or differ at most by one;
  (2) At any point, the mean value of the envelope defined by the local maxima and the envelope defined by the local minima is zero.
2. The EMD decomposition of the ith ping echo intensity $X^{i}$ is according to the following steps:
  (1) Initialization: $r_{s0}=X^{i},p=1$;
  (2) Computation of the pth IMF, $IMF_{sp}$:
    (a) Initialization: $h_{0}=r_{s(p-1)},\tilde{j}=1$
    (b) Find the local maxima and minima of $h_{\tilde{j}-1}$
    (c) Interpolate the local minima (resp. maxima) to get $e_{1}^{}$ (resp $e_{2}^{}$) by cubic spline
    (d) Compute the mean of these envelops: $E=\left(e_{1}^{}+e_{2}^{}\right)/2$
    (e) Obtain the $\tilde{j}$th component $h_{\tilde{j}}^{}$ by $h_{\tilde{j}}^{}=h_{\tilde{j}-1}-E^{}$
    (f) If the above two conditions of IMF are satisfied, then $IMF_{sp}=h_{\tilde{j}}$; else, $\tilde{j}=\tilde{j}+1$;
  (3) Calculate residual component $r_{sp}^{}=r_{s(p-1)}-IMF_{sp}$;
  (4) If $r_{sp}^{}$ is not monotonic, go to step (2); otherwise, the decomposition is complete.

When the decomposition is complete, we can write the ith ping echo intensity $X^{i}$ as $X^{i}=\sum_{p=1}^{P}IMF_{sp}^{}+r_{sP}^{},P\in {\mathbb{N}}^{*}$.
